# Prognostic value of lymph node involvement in oral squamous cell carcinoma

**DOI:** 10.1007/s00784-022-04630-7

**Published:** 2022-07-27

**Authors:** Jan Oliver Voss, Lea Freund, Felix Neumann, Friedrich Mrosk, Kerstin Rubarth, Kilian Kreutzer, Christian Doll, Max Heiland, Steffen Koerdt

**Affiliations:** 1 Charité - Universitätsmedizin Berlin, corporate member of Freie Universität Berlin and Humboldt-Universität zu Berlin, Department of Oral and Maxillofacial Surgery, Augustenburger Platz 1, 13353 Berlin, Germany; 2 Berlin Institute of Health (BIH), Anna-Louisa-Karsch-Straße 2, 10178 Berlin, Germany; 3 Charité - Universitätsmedizin Berlin, corporate member of Freie Universität Berlin and Humboldt-Universität zu Berlin, Institute of Biometry and Clinical Epidemiology, Charitéplatz 1, 10117 Berlin, Germany; 4 Charité - Universitätsmedizin Berlin, corporate member of Freie Universität Berlin and Humboldt-Universität zu Berlin, Institute of Medical Informatics, Charitéplatz 1, 10117 Berlin, Germany

**Keywords:** Oral squamous cell carcinoma, Selective neck dissection, Modified radical neck dissection, Lymph node metastasis, Disease-free survival, Overall survival

## Abstract

**Objectives:**

Different parameters have been identified in patients with oral squamous cell carcinomas (OSCC) that have a serious impact on survival, including residual tumour and extracapsular spread. Moreover, other factors, including the lymph node ratio (LNR) and lymph node yield (LNY), have been suggested as prognostic markers.

**Material and methods:**

This retrospective study included patients diagnosed with OSCC and cervical lymph node metastases during the years 2010–2020. Patients’ records were evaluated regarding lymph node status, final therapy regime, tumour recurrence, time to death, tumour association with death, disease-free survival (DSF), and overall survival (OS).

**Results:**

In 242 patients with a mean age of 63.57 ± 11.24 years, treated either by selective neck dissection (SND; *n* = 70) or by modified radical neck dissection (MRND; *n* = 172), 5772 lymph nodes were detected. The LNR and LNY were identified as independent risk factors in OS and DFS. The optimal cut-off point for the LNY was ≥ 17 lymph nodes in the SND and ≥ 27 lymph nodes in the MRND group.

The metastatic lymph node clearance (MLNC) was established as a score to relate the LNR and LNY to the extent of lymph node removal. Survival analysis showed statistically significant differences among score levels.

**Conclusions:**

As information about the extent of nodal dissection is excluded from LNR and LNY, we propose the use of a new scoring system comprising individual cut-off values for LNY and LNR with regard to the extent of neck dissection.

**Clinical Relevance:**

MLNC might help to identify high-risk OSCC patients with metastatic lymph nodes.

## Introduction

Cancers of the oral cavity, of which more than 90% are oral squamous cell carcinomas (OSCCs), account for approximately 30% of all head and neck cancers [1, 2]. In the case of locally advanced disease and in the absence of distant metastases, surgical resection is considered the gold standard in oncologic therapy. Nevertheless, the involvement of cervical lymph nodes (LN) (N-status) is among the most important independent prognostic factors in OSCC [3–5]. Due to a relatively high number of occult LN metastases, nodal clearance represents an important factor in surgical head and neck oncology. Different types of neck dissections (ND) adapted to preoperative staging of the neck and different algorithms are in clinical use [6]. However, not only the N-status of the disease but also other LN parameters could be identified as prognostic factors in overall (OS) and disease-free survival (DFS) analysis of OSCC. Besides the total number of metastatic lymph nodes (MLN), contralateral LN status, location of LN metastases according to the levels of Robbin, and extracapsular spread (ECS) are also considered to play important prognostic roles [7]. 

In other entities, such as colorectal carcinoma, bladder cancer, breast cancer, and oesophageal cancer, the lymph node ratio (LNR), also known as lymph node density (LND), calculated as the number of positive nodes relative to the total number of examined nodes, has been established as an independent prognostic factor for OS and DFS [8–11]. Data on the value of parameters such as the LNR in long-term follow-up of OSCC describe poor outcomes in patients with a comparably high LNR [12–17]. Patel et al. found a LNR below 7% to be associated with significantly increased OS in OSCC [16]. Moreover, they showed that the combination of the TNM-staging system with LNR was superior to the TNM system alone. However, the main concern regarding the informational value of the LNR is the variability in the ratio within the same patient treated by different types of ND.

Apart from the LNR, other parameters, such as extracapsular spread (ECS) status, and the total number of removed LN (lymph node yield, LNY), depending on the type of ND, have been analysed in the literature, and their prognostic value has been described [18–20].

ECS has been identified in numerous studies, for example, the analysis by Michikawa and colleagues, as one of the most important predictors of a poor treatment outcome [21]. ECS is one important factor in the decision to intensify adjuvant therapy, as the prognosis of ECS in OSCC patients is associated with the level of progression of ECS [22]. ECS accounts for significantly reduced OS and DFS, and the presence of ECS in patients with OSCC generally indicates a poor overall prognosis [23].

In general, the extent of the ND impacts the total number of LN removed. Different reports have been published lately with different conclusions regarding the required total number of removed LN in selective neck dissection (SND) and overall survival [24–26]. Besides clearance control, the time point of LN removal has also been analysed. In a prospective, randomized controlled trial, patients treated with an elective ND (neck dissection at the time of the primary surgery) in T1 and T2 oral squamous-cell carcinoma showed higher rates of overall and disease-free survival than those treated with a therapeutic ND after nodal relapse [27].

One concern when comparing LNR of different patients is that the underlying type of neck dissection is not considered. Therefore, the information about the LRN could be misleading.

The current retrospective study aims to evaluate the prognostic value of clinical and histopathological aspects of LN metastases in OSCC and to correlate these findings to the long-term follow-up. A new scoring system is introduced, which considers LNR and LNY in the clinical setting.

## Material and methods

### Ethical agreement.

The institutional review board of the Charité—Universitätsmedizin Berlin gave ethical approval for data collection and publication (EA1/077/20).

### Study design

This study represents a retrospective analysis of all patients with OSCC who were treated in the Department of Oral and Maxillofacial surgery of the Charité—Universitätsmedizin, Berlin, Germany during the years 2010–2019. All included patients were treated in a curative stetting and showed LN metastases in histological work-up (pN +). Treatment approaches were decided by the interdisciplinary tumour board based on the National Comprehensive Cancer Network practice guidelines in Head and Neck Cancers [28]. Electronic and paper-based patient records were further evaluated regarding age, gender, age at the time of malignancy diagnosis, daily alcohol and/or nicotine abuse (including pack years), results of histological analysis, clinical and pathological TNM classification, clinical and pathological stadium based on the Union for International Cancer Control (UICC 8th edition, 2017), LN status (including total number removed, total number of cervical LN metastases, localization (including side of the neck), affected Robbins Level (I–V) and extracapsular spread), final therapy regime (surgery, chemotherapy, radiation or combination therapy), substance of adjuvant chemotherapy, localization and doses of radiation (anatomical region of primary tumour and/or regional lymphatic pathways), recurrence of tumour, regional and/or remote metastasis as well as total number of each, time to death as well as tumour/cancer-related death, disease-free survival, and overall survival. However, only patients with complete and comprehensive datasets were included in this retrospective study. OS was defined as the time between surgical tumour removal and death or last follow-up. DFS was defined as the time between surgical tumour removal and tumour recurrence, the occurrence of metastasis, death, or the last follow-up. All dates of death were collated with the population registry.

### Data analysis

The data were collected in Microsoft Excel (Microsoft Corporation, Redmond, WA, USA) and analysed by using SPSS Statistics Version 27.0 (IBM Corporation, Armonk, NY, USA). ND including levels I–III (uni- or bilateral) were classified as SND, and ND including levels I–V were classified as modified radical neck dissection (MRND) (with or without removal of other muscular, nervous, or vascular structures). In all patients, surgical treatment including neck dissection was performed according to current guidelines. Recommendations for adjuvant radiation or chemoradiation were based on the interdisciplinary tumour board decision.

In cases of bilateral ND with MLN on only one side of the neck, only the side of the neck with the metastasis was included in the analysis. In cases of bilateral ND with a differing extent of LN removal between the two sides (SND vs. MRND), only the MRND side was evaluated. In cases of bilateral ND with MLN on both sides and the same extent of LN removal (SND or MRND), the mean value for both sides was used. For further evaluation, a subgroup comparison between SND and MRND was performed.

LNY refers to the total number of removed LN on one side of the neck and was specified separately for SND and MRND. LNR is defined as the ratio of the number of MLN to the total number of LN removed and was also specified separately for SND and MRND. Means and standard deviations (SD) for continuous data and medians and 1^st^ and 3^rd^ quartiles for categorical data were calculated. Cox regression analysis was performed for LNY and LNR; the models were adjusted for age, sex, ECS status, and pathological tumour status (T-status); and hazard ratios (HR) were calculated. Kaplan–Meier analyses were performed for survival analysis, calculating OS and DFS. Log-rank tests were performed to test for relationships between categorical variables and OS or DFS. Cut-offs were calculated by using ROC curves based on logistic regression, since data were reconciled with the population registry to ensure that censored patients were still alive at the study end. The optimal cut-off was defined via Youden’s index. The results were considered statistically significant at *p* < 0.05. Due to the exploratory nature of the study, no adjustment for *p* values was applied. Therefore, the results of statistical tests should be interpreted as exploratory, not confirmatory.

## Results

### Patient characteristics

A total of 242 patients were included in this retrospective study. Approximately one-third were female (*n* = 86, 35.5%), and 64.5% were male patients (*n* = 156). The mean patient age was 63.57 ± 11.24 years (min = 34, max = 94).

In terms of risk factors, 94 patients (38.8%) used neither alcohol nor tobacco. Sixty-six patients (27.3%) reported being smokers, whereas alcohol abuse was only present in nine (3.7%) patients. About one-third of all patients reported using both alcohol and tobacco on a daily basis (*n* = 73, 30.2%). Pack years were available for 62 of the 139 patients (32.1%). On average, this group had a smoking history of 45.74 ± 21.85 pack years (min = 10, max = 100). Localizations of OSCCs are described in Table 1.

**Table 1:** Detailed information regarding tumour localization and number of patients

Localization	*n* (%)
Floor of mouth	74 (30.6)
Tongue	65 (26.9)
Upper gum	9 (3.7)
Lower gum	40 (16.5)
Others (cheek, vestibule, or retromolar area)	42 (17.4)
Palate	10 (4.1)
Overlapping lesions of the lip, oral cavity, and pharynx	2 (0.8)

In total, 212 (87.6%) of the tumours were located unilaterally: 91 (37.6%) on the right side and 121 (50.0%) on the left side. Moreover, tumour location was bilateral in 4 patients (1.7%) and anterior in 26 (10.7).

SND was performed in 70 patients, while 172 patients received a MRND. In total, 77 patients were initially staged with a clinically negative neck (cN −), while 165 patients were staged with a clinically positive neck (cN +). Histopathological examination detected 5772 LN in 242 patients. Overall, in each neck dissection, there were 3–78 (median = 23.00, q1 = 15.00, q3 = 29.00) LN and 1–13 (median = 2.00, q1 = 1.00, q3 = 3.00) metastases. The total number of removed LN in SND (*n* = 1708, median = 24.00, q1 = 17.00, q3 = 29.00) was smaller than that in MRND (*n* = 4064, median = 22.00, q1 = 14.00, q3 = 28.00). The overall rate of occult metastasis in cN − patients was 31.8%. There were 82 patients with pN1 disease (33.9%), 76 patients with pN2 disease (31.4%), and 84 with pN3 disease (34.7%). Clinical and pathohistological TNM and UICC status are summarized in Table 2.

**Table 2:** Clinical and pathohistological TNM and UICC status

Parameter	*n* (%)	Parameter	*n* (%)
cT stage		pT stage	
cT0	5 (2.1)		
cT1	47 (19.4)	pT1	37 (15.3)
cT2	104 (43.0)	pT2	89 (36.8)
cT3	17 (7.0)	pT3	44 (18.2)
cT4a	69 (28.5)	pT4a	70 (28.9)
		pT4b	1 (0.4)
		pTx	1 (0.4)
cN stage		pN stage	
cN0	77 (31.8)		
cN1	59 (24.4)	pN1	82 (33.9)
cN2a	31 (12.8)	pN2a	24 (9.9)
cN2b	38 (15.7)	pN2b	40 (16.5)
cN2c	37 (15.3)	pN2c	12 (5.0)
		pN3b	84 (34.7)
cM stage		pM stage	
cM0	228 (94.2)	pM0	239 (98.8)
cM1	8 (3.3)	pM1	3 (1.2)
cMx	6 (2.5)		
cUICC		pUICC	
0	3 (1.2)	0	
I	26 (10.7)	I	
II	31 (12.8)	II	
III	48 (19.8)	III	70 (28.9)
IVa	119 (49.2)	IVa	86 (35.5)
IVb	7 (2.9)	IVb	82 (33.9)
IVc	8 (3.3)	IVc	3 (1.2)
		n/a	1 (0.4)

*Abbreviations:n/a*, not applicable

### Survival and follow-up

Median follow-up from the time of treatment to the last visit or the patient´s death was 24.48 months (0.66–118.93, q1 = 10.95, q3 = 49.29) and 35.35 months (0.69–118.93, q1 = 17.31, q3 = 69.72) of surviving patients. In total, 179 patients (74%) received adjuvant therapy.

### Extracapsular spread (ECS)

ECS was detected in 109 patients (45.0%). ECS was almost identical in MRND (*n* = 77, 44.8%) and in SND (*n* = 32, 45.7%). Assessment of follow-up in terms of OS and DFS showed significant differences between ECS-positive and ECS-negative cases (OS: *p* < 0.001; DFS: *p* < 0.001).

### Lymph node ratio (LNR)

The median LNR was 0.08 (range: 0.02–0.67, q1 = 0.05, q3 = 0.14). HR for OS (7.06; *p* < 0.01; 95% CI = 1.92–25.94) and DFS (8.22; *p* < 0.01; 95% Cl = 2.37–28.49) were calculated. Thereby, LNR was identified as an independent risk factor, referring to OS and DFS. Furthermore, older age, advanced pT stage, and ECS were determined as independent risk factors for poorer OS and DSF. All results of Cox regression are displayed in Table 3.

**Table 3:** Results of Cox regression model for LNR for OS and DFS

	Parameter	*p*-value	HR	95% CI
OS	Sex	0.79	0.95	0.64–1.41
	Age	< 0.001	1.03	1.02–1.05
	pT	0.04	1.19	1.01–1.42
	ECS	< 0.001	2.90	1.93–4.35
	LNR	0.003	7.06	1.92–25.94
DFS	Sex	0.54	0.89	0.63–1.28
	Age	0.001	1.03	1.01–1.04
	pT	0.05	1.17	1.00–1.36
	ECS	< 0.001	2.05	1.43–2.95
	LNR	0.001	8.22	2.37–28.49

For determination of ideal cut-off points, ROC analysis was performed using logistic regression. Optimal cut-off points for DFS analysis in the SND and MRND groups were determined using the Youden index.

**Fig. 1:**
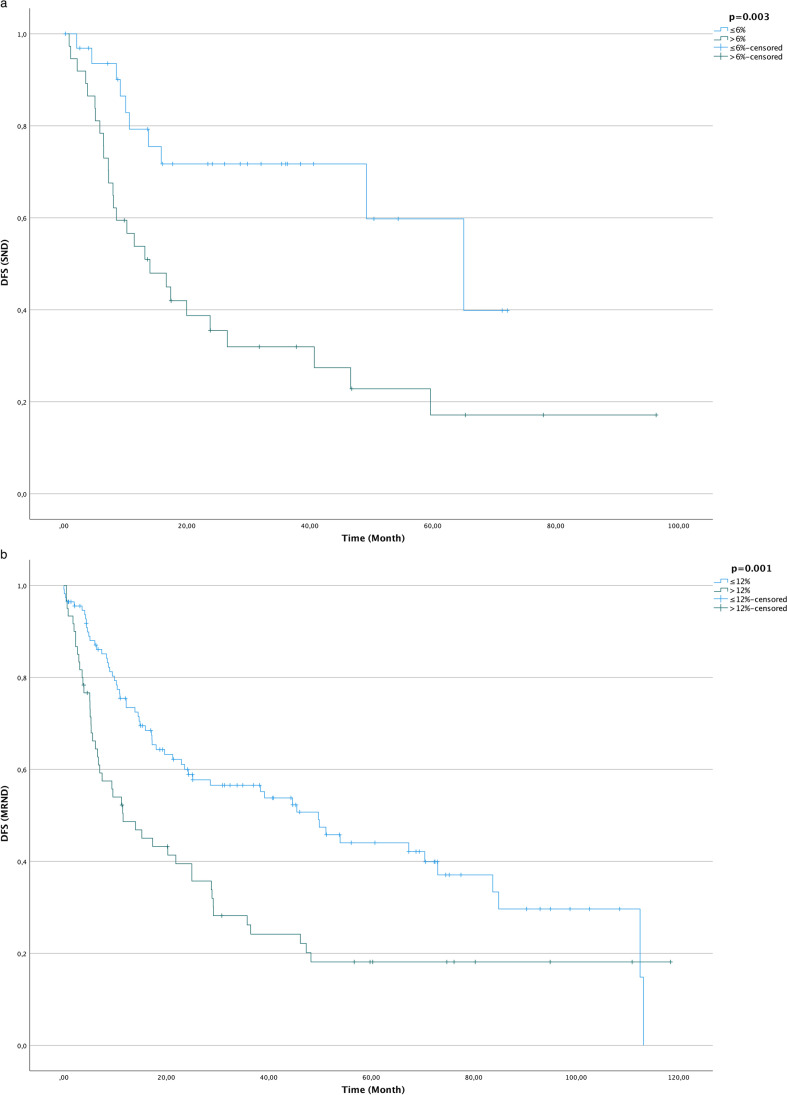
Kaplan–Meier curves for disease-free survival (DFS; time in months) with regard to lymph node ratio (LNR): **a** selective neck dissection with a LNR threshold ≤ 0.06 and **b** modified radical neck dissection with a LNR threshold ≤ 0.12

For the SND group, the optimal cut-off point for DFS was a LNR ≤ 0.06 (AUC = 0.71; CI = 0.58–0.83, SD = 0.07, *p* < 0.01) with a sensitivity of 73% and a specificity of 70%. For the MRND group, the cut-off point for DFS was LNR = 0.12 (AUC = 0.65; CI = 0.57–0.73, SD = 0.04, *p* ≤ 0.01) with a sensitivity of 44% and a specificity of 79%. DFS (*p* ≤ 0.01) showed statistically significant differences between LNR groups; the results are displayed graphically in Fig. 1.

### Lymph node yield (LNY)

HR for LNY were calculated for OS (0.98; *p* = 0.01; 95% CI = 0.96–1.00) and DFS (0.98; *p* < 0.01; 95% CI = 0.96–0.99). Thus, LNY could be identified as an independent risk factor for OS and DFS. Also, older age, advanced pT stage, and ECS were determined as independent risk factors for poorer OS and DFS. All results of Cox regression analysis are summarized in Table 4.

**Table 4:** Results of a Cox regression model for lymph node yield for overall survival and disease-free survival

	Parameter	*p*-value	HR	95% CI
OS	Sex	0.59	0.90	0.61–1.33
	Age	≤ 0.001	1.04	1.02–1.05
	pT	0.02	1.23	1.04–1.46
	ECS	< 0.001	3.17	2.15–4.68
	LNY	0.01	0.98	0.96–1.00
DFS	Sex	0.35	0.85	0.60–1.20
	Age	≤ 0.001	1.03	1.01–1.05
	pT	0.02	1.21	1.04–1.41
	ECS	< 0.001	2.22	1.57–3.16
	LNY	0.004	0.98	0.96–0.99

ROC analysis was performed to calculate the ideal cut-off point. The optimal cut-off point for the SND group was ≥ 17 LN referring to DFS (AUC = 0.61; 95% CI = 0.48–0.74, SD = 0.07, *p* = 0.11) with a sensitivity of 82% and a specificity of 38%.

**Fig. 2:**
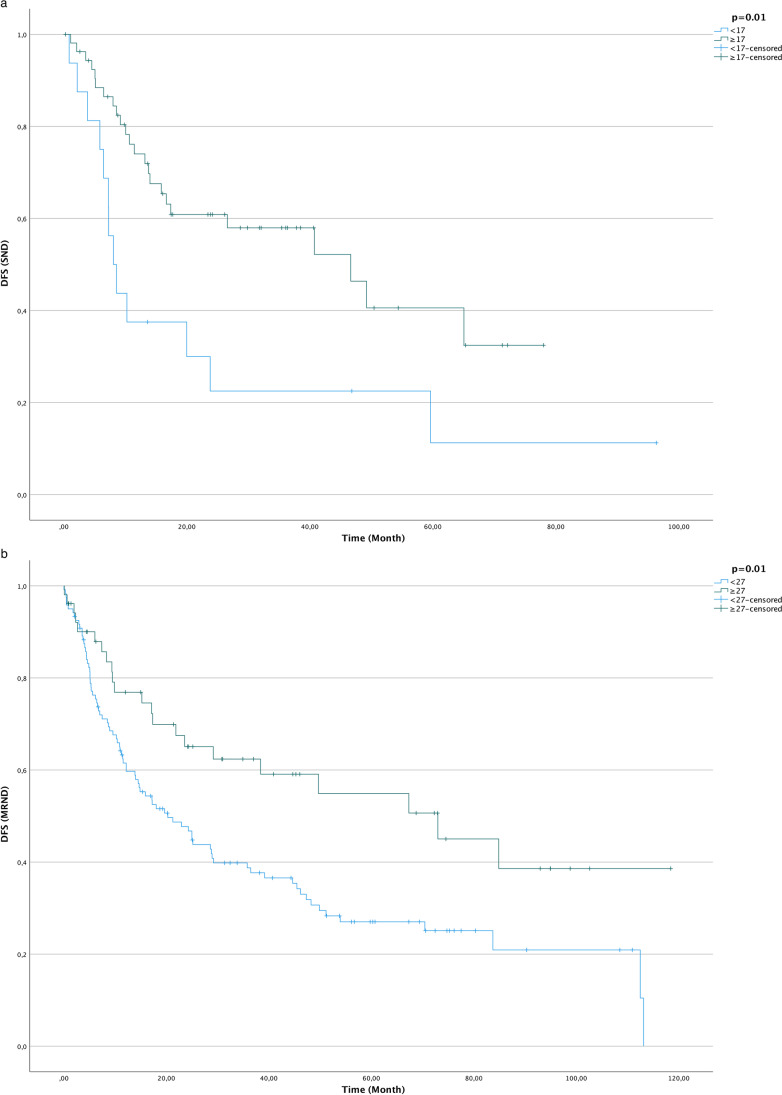
Kaplan–Meier curves for disease-free survival (time in months) with regard to lymph node yield (LNY): **a** selective neck dissection with a LNY threshold ≥ 17 and **b** modified radical neck dissection with a LNY threshold ≥ 27

The optimal cut-off point for DFS in the MRND group was ≥ 27 LN (AUC = 0.64; 95% CI = 0.56–0.73, SD = 0.04, *p* ≤ 0.01) with a sensitivity of 40% and a specificity of 81%. In the SND group, 17 or more LN were dissected in a total of 77.1% (*n* = 54) cases, whereas 22.9% of all patients (*n* = 16) who received a SND had fewer than 17 LN in pathohistological analysis. In the MRND group, 52 patients received ND with 27 or more LN dissected (30.2%), whereas 120 patients received a ND with fewer than 27 LN in pathohistological analysis (69.8%). Kaplan–Meier analysis showed statistically significant differences in DFS in both the SND (*p* = 0.01) and the MRND groups (*p* = 0.01) (Fig. 2).

### Metastatic lymph node clearance (MLNC)

The metastatic lymph node clearance (MLNC) was established as a score to relate LNR and LNY in the clinical setting, as the total number of removed LN differs between SND and MRND, which should be taken into account. Point values 0 and 1 were matched to LNR and LNY cut-off values. Point values were summed to obtain a total score (min = 0 and max = 2). Table 5 displays the allocation of LNY and LNR to point values in SND and MRND.

**Table 5:** Metastatic lymph node clearance (MLNC)

SND	LNY		LNR	
	≥17	1	≤0.06	1
	<17	0	>0.06	0
MRND	LNY		LNR	
	≥27	1	≤0.12	1
	<27	0	>0.12	0

**Fig. 3:**
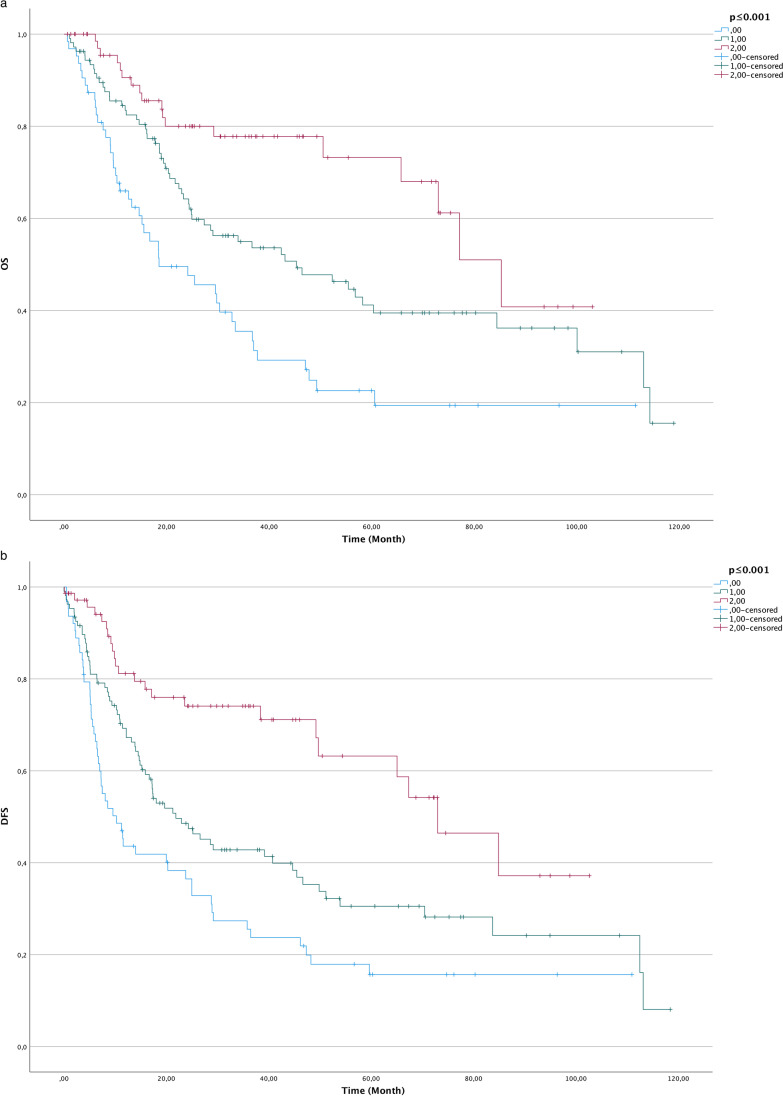
Kaplan–Meier curves for overall survival (OS; **a**) and disease-free survival (DFS; **b**) in groups regarding the metastatic lymph node clearance score (time in months)

Kaplan–Meier analysis was performed for the evaluation of different score levels regarding OS and DFS. Log-rank tests showed statistically significant differences in OS (*p* ≤ 0.001) and DFS (*p* ≤ 0.001) among the score levels (Fig. 3).

## Discussion

Surgical treatment of patients with OSCC includes both radical resection of the primary tumour and removal of neck LN to different extents depending on the results of clinical and radiological examinations, due to the risk of occult nodal metastasis [29]. The American Joint Committee on Cancer (AJCC) system stages head and neck cancer patients systematically on the basis of the TNM classification system. This staging system summarizes the nodal information, including number, size, ECS status, and neck side of positive LN, conclusively [30].

Different information are derived from ND analysis: the regional disease spread (total number of metastatic cervical lymph nodes) and the surgical extension (total number of removed lymph nodes). However, besides the quality of the neck dissection itself (surgeon’s accuracy at clearing the levels of the neck), the sampling factor (the completeness of the pathological analysis) influences the probability of identifying metastases in LN [31, 32]. Therefore, the value of the ND is influenced by the surgical and histopathological accuracy/performance.

In general, nodal disease is associated with a poor outcome and is among the most important independent prognostic factors in head and neck carcinomas [33–36]. Besides nodal stage, resection margins as well as ECS are significant prognostic factors for both loco-regional recurrence and survival in patients with high-risk cancers of the oral cavity, oropharynx, larynx, or hypopharynx [37].

The total number of MLN has been reported as a superior predictor of survival in OSCC patients in comparison to the AJCC N-staging system [38]. However, as limited LN dissection and detection might lead to nodal understatement, different additional valuation parameters have been proposed, including LNY and LNR [39, 40].

LNY has been reported as an independent prognostic factor in OSCC patients undergoing elective neck dissection [41]. Interestingly, while a nodal yield ≥ 18 was associated with better overall survival than a LNY < 18, this effect was not linear, as resection of more than 32 LN had a negative effect on survival [39, 42]. As the non-linear relationship between nodal yield and overall survival is transferred to the calculation of LNR, previous authors have questioned the prognostic value of the LNR [7, 38].

The LNR aims to consider different factors that potentially influence nodal staging: (1) tumour-specific factors (the total number of positive lymph nodes), (2) surgical factors (number of LN removed during neck dissection), and (3) histological factors (the completeness of the pathological analysis) [16].

The LNR has previously been reported as an independent prognostic factor in OSCC [43, 44]. In a meta-analysis, a high LNR was significantly related to short OS, DSS, and DFS [45]. In a multicentre study, Patel et al. investigated the LNR in oral cavity cancer and concluded in a multivariate analysis that a LNR smaller than 0.07 was associated with better local control, loco-regional control, and DFS. Moreover, Patel and colleagues provided evidence that a new LNR-based TNM staging system is superior to the traditional staging system in estimating survival measures, including OS, DSS, and locoregional control [16]. However, in a review of the literature, Talmi and colleagues concluded that in order to transfer the information obtained from the LNR into treatment modification, more precise, prospective randomized trials are required [46].

However, calculating the LNR without considering the underlying type of neck dissection carries the risk of diluting the ratio due to the higher number of total resected lymph nodes. In this regard, Locca and colleagues found, in a multiple regression analysis, that LNY statistically significantly predicted the LNR [47]. Moreover, one limitation of using the LNR is that categorization of patients with the same LNR is not sufficient, as a LNR of “1” could possibly represent one positive LN in one LN resected (1/1) or 20 positive LN in 20 total resected (20/20). Gleisner and colleagues analysed patients with colon cancer retrospectively and concluded that the LNR does not properly represent the prognostic significance of the total number of positive LN and the LNY among patients with colon cancer [48].

To our understanding, it is inevitable to (a) consider/distinguish the extent of neck dissection, (b) consider both the total number of metastatic LNs and the total number of LNs, and (c) correlate the LNR and LNY to specific cut-off values, which then sum to a conclusive score. We propose the use of a scoring system that considers the cut-off values for both LNY and LNR separately for SND and MRND, which is called the metastatic lymph node clearance (MLNC). In this study, survival analysis showed statistically significant differences in OS and DFS among the score levels of MLNC. While the AJCC staging system considers neither the total number of LN removed nor the intended extent of LN removal, the MLNC combines this information into a comprehensive score. Furthermore, by using the MLNC score instead of the LNR and LNY, information about the corresponding cut-off values is considered as well. However, randomized prospective trials are indispensable to proving the prognostic informative value of the MLNC score and retrieving therapeutic recommendations.
